# Hemorrhagic liver cyst misdiagnosed as echinococcosis in a Tibetan woman: A case report

**DOI:** 10.1097/MD.0000000000040940

**Published:** 2024-12-27

**Authors:** Shuang Wang, Yan He, Xuchang He, Jun Wang

**Affiliations:** a Department of Hepatobiliary and Pancreatic Surgery, Chengdu Fifth People’s Hospital, Chengdu, Sichuan, PR China; b School of Medical and Life Sciences, Chengdu University of Traditional Chinese Medicine, Chengdu, Sichuan, PR China.

**Keywords:** echinococcosis, imaging examination, intracystic hemorrhage, liver cystic

## Abstract

**Rationale:**

We describe a case of hepatic cyst with intracystic bleeding that was preoperatively misdiagnosed as hepatic echinococcosis. Pain in the right upper quadrant of the abdomen was the first symptom to appear. In addition to highlighting the significance of imaging in distinguishing hepatic cyst with intracystic bleeding from other cystic liver tumors, we also talk about the difficulties and obstacles encountered throughout the diagnosis and treatment of this case. More imaging evidence for the diagnosis of hepatic cyst with intracystic bleeding is anticipated in this patient.

**Patient concerns:**

A 24-year-old Tibetan female was admitted to the hospital due to swelling and pain in the right upper abdomen for more than 1 month.

**Diagnoses:**

The patient had a history of living in the epidemic area, serum echinococcosis antibody was positive, and abdominal computed tomography plain enhanced scan showed a low-density cystic space-occupying lesion in the right lobe of the liver, with a high-density mass in the cyst, which was misdiagnosed as cystic hepatic echinococcosis before operation. Postoperative pathological examination confirmed old hemorrhage in hepatic cysts, and no parasitic infectious lesions such as *Echinococcus granulosus* were found.

**Interventions:**

After perfecting the relevant preoperative preparation, the patient underwent right hemihepatectomy plus portal vein and vena cava repair.

**Outcomes:**

The patient was discharged from the hospital 5 days after operation.

**Lessons:**

The differential diagnosis of individuals with a history of residing in an epidemic region and liver space-occupying lesions on imaging should take into account more than only the probability of hepatic echinococcosis, as this case highlights. When making a differential diagnosis of different types of cystic liver tumors, imaging testing is crucial.

## 1. Introduction

As the most common benign neoplastic lesion of the liver, hepatic cysts contain a clear fluid in their cysts, ranging from a few millimeters to tens of centimeters in diameter. Most simple hepatic cysts are incidentally detected by ultrasound or computed tomography (CT) during physical examination.^[1]^ Because hepatic cysts are usually asymptomatic, they do not require treatment, except when there are severe complications. Unlike smaller cysts, larger cysts are more prone to complications such as bleeding, rupture, infection, and biliary tract compression.^[2]^ Mass effect and compression of adjacent structures are the most common complications. Most patients present with abdominal pain, and in rare cases, intracapsular hemorrhage can be complicated.^[3]^ When liver cysts contain large amounts of blood clots, it is difficult to differentiate such lesions from cystic echinococcosis or cystic liver tumors on diagnostic imaging.^[4]^ After an extensive literature search, we found that hepatic cysts with hemorrhage have not been misdiagnosed as cystic hepatic echinococcosis. We hope that this case highlights the importance of imaging in the diagnosis of hepatic cysts with hemorrhage, even in patients with a clear history of travel to the epidemic area and positive specific serum antibodies. Written informed consent was obtained from the patients, and the surgical case report criteria was met.

## 2. Case report

**Figure 1.**
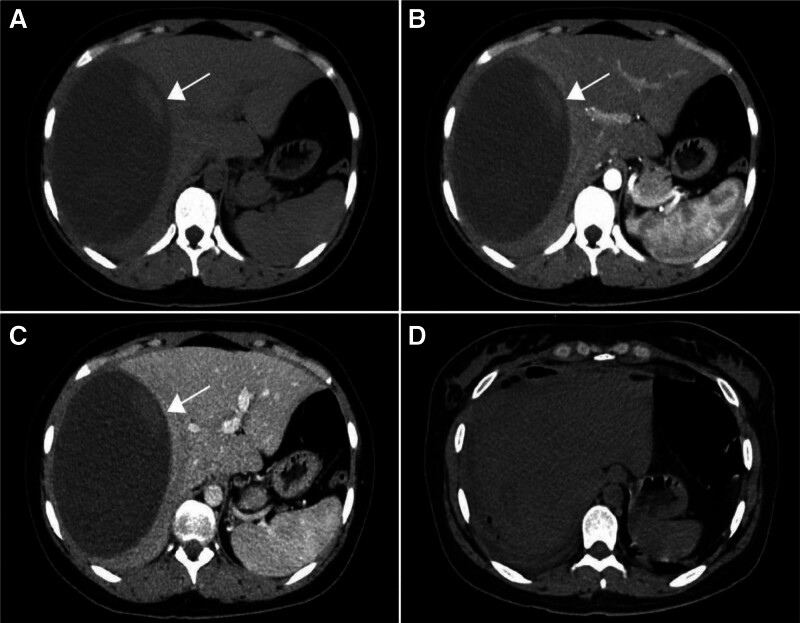
The preoperative plain and enhanced CT images of the patients and the abdominal CT images of the patients were reviewed at 4 months after surgery. (A) CT plain scan showed a 14 × 11.3 cm cystic lesion in the right lobe of the liver, with high-density mass shadow in the lesion. (B) There was no obvious enhancement of the lesion and cyst contents in the arterial phase of CT enhancement. (C) Contrast-enhanced CT showed no significant enhancement of the lesion and cyst contents in the portal vein phase. (D) Four months after the operation, the CT scan showed no obvious abnormality.

**Figure 2.**
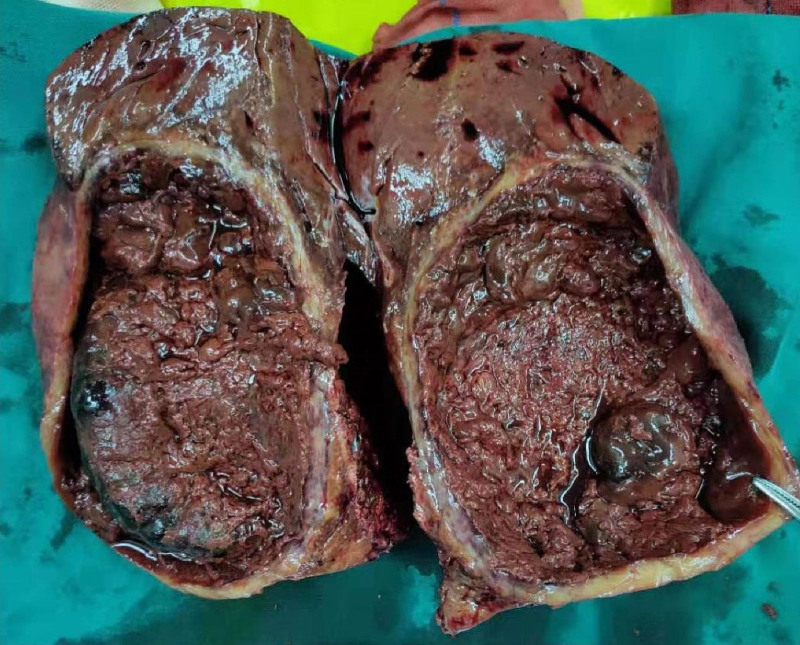
On examination of the specimen, the wall of the giant cyst was significantly thickened, and its internal surface was rough and filled with chocolate-colored fluid.

A 24-year-old female patient was admitted to the hospital due to swelling and pain in the right upper abdomen for >1 month. The pain in the right upper quadrant was not severe, accompanied by pain and discomfort in the right shoulder. The patient denied any gastrointestinal symptoms such as nausea, vomiting, constipation, and diarrhea, as well as fever, chills, and jaundice of the skin and sclera. The patient had not taken any treatment for the relief of her symptoms before he presented to our hospital. In addition, it is worth noting that the patient came from the Tibetan area of western Sichuan, which is one of the endemic areas of echinococcosis in China, and had a clear history of living in the endemic area. The rest of the medical history and family history were normal. Because the patient had lived in an endemic area of hepatic echinococcosis for many years, she expressed concern to us during the course of the history taking that echinococcosis might be the cause of her current illness. Physical examination revealed a slight bulge in the right upper quadrant with mild tenderness, without rebound or muscle tension. The liver was enlarged, and the lower edge of the liver was palpable. Contrast-enhanced computed tomography of the abdomen revealed a 14 × 11.3 cm cystic lesion in the right lobe of the liver with a high-density mass within it and no significant enhancement (Fig. 1A–C).The complete blood count, electrolytes, and eosinophil count were normal, alanine aminotransferase and alkaline phosphatase were slightly elevated, albumin was slightly decreased, and alpha-fetoprotein, carcinoembryonic antigen, and serum carbohydrate antigen were within normal limits. Results of stool parasitology and white-cell testing were negative. However, a serologic test for echinococcus antibodies in the patient was positive for anti-echinococcus immunoglobulin G antibodies. Therefore, after considering the patient’s symptoms, history of travel to endemic areas, imaging findings, and serologic test results for echinococcus antibodies, we highly suspected that the patient had cystic hepatic echinococcosis. After completing all preoperative preparations and a thorough assessment of surgical risks, we prepared the patient for right lobectomy of the liver and repair of the portal vein and vena cava. During the operation, we found a large cystic lesion in the right liver lobe that was tightly adhered to the diaphragm. Dissection of the excised giant cyst revealed a rough internal surface (Fig. 2) that was filled with dark chocolate fluid. Postoperative pathological examination confirmed that the effusion was a hepatic cyst with old hemorrhage in the cyst, and no *Echinococcus granulosus* (*E granulosus*) and other parasitic infectious lesions were found. Inflammatory infiltration around the cyst wall contained a large amount of fibrotic scar tissue. The patient was discharged healthy 5 days after surgery, and we found no evidence of echinococcosis when we followed up the patient’s abdominal CT scan 4 months after surgery (Fig. 1D). Due to the limitation of her own economic conditions, the patient did not return to our medical institution after the reexamination 4 months after the operation. We learned by telephone that the patient recovered well after the operation, and no evidence of hepatic echinococcosis infection was found.

## 3. Discussion

Simple hepatic cysts are typically cystic, thin-walled masses with fluid-filled epithelial lining lacunae. It is often caused by abnormal development of the bile duct during the embryonic period. Its incidence ranges from 2.5% to 18% and increases with age.^[2]^ The cyst wall is lined with epithelial cells that secrete clear fluid. Congenital hepatic cysts grow slowly and vary in size. Small hepatic cysts are usually asymptomatic; however, the appearance of a compression effect as a hepatic cyst grows can cause abdominal discomfort, pain, bloating, and gastrointestinal symptoms such as nausea, vomiting, fullness, and early satiety. When the cyst grows to a certain extent, a palpable abdominal mass may appear. The common complications of simple hepatic cysts include infection, spontaneous hemorrhage, rupture, and external compression of the biliary tree and major blood vessels.^[5]^ It should be noted that in some simple hepatic cysts, due to excessive pressure in the cyst cavity, necrosis of the vascular epithelial tissue of the cyst wall may occur and lead to the occurrence of intracystic hemorrhage.^[6]^ The main clinical manifestations of hepatic cysts with intracystic hemorrhage were acute or chronic abdominal pain in most patients, rapid increase in cystic mass volume and jaundice during regular examination.^[7]^ As a rare complication of hepatic cysts, intracystic hemorrhage is not common in clinical practice.^[8]^ At present, congenital hepatic cysts with intracystic hemorrhage can be treated nonoperatively or surgically. The nonsurgical method is mainly percutaneous puncture drainage, which is mainly used for patients with poor general conditions or elderly patients. The recurrence rate of nonsurgical treatment is relatively high. Some studies have reported a recurrence rate of up to 100% after percutaneous puncture and drainage. Surgical procedures included cyst fenestration, liver resection, and liver transplantation. Liver resection should be considered if cystic hepatic echinococcosis, cystadenoma, or cystadenocarcinoma cannot be ruled out. Liver transplantation is suitable for patients with poor liver function, who cannot tolerate surgery, and those with small residual functional liver volume.^[9]^

Simple hepatic cysts have typical imaging manifestations and are easy to be diagnosed clinically, but the imaging manifestations of hepatic cysts with intracystic hemorrhage are complex. Intracystic hemorrhage showed changes in cyst density, blood stratification in the cyst, or diffuse irregular hyperecho on imaging.^[10]^ Several neoplastic lesions of the liver can be confused with hepatic cysts with hemorrhage, including biliary cystadenoma, cystadenocarcinoma, and primary embryonal sarcoma.^[11]^ According to clinical and imaging data, it is sometimes difficult to distinguish hemorrhagic hepatic cysts from cystic malignant tumors when mural nodules with enhancement after intravenous administration of contrast material appear on CT or magnetic resonance imaging (MRI) examination.^[6,12]^ It is also worth to note that hemorrhagic cystic lesions are hyperintense on T1-weighted images, nonhemorrhagic cystic lesions are hypointense on T1-weighted images, and both cystic lesions are hyperintense on T2-weighted images. Nonhemorrhagic cysts are typically hypodense on CT, whereas hemorrhagic cysts can appear hyperdense in the acute phase and hypodense or mixed density after the acute phase.^[9]^ All of the above conditions may lead to a variety of changes in the imaging picture of simple hepatic cysts. These alterations make it difficult to diagnose hepatic cysts with hemorrhage on imaging with other liver diseases such as hydatid disease, biliary cystadenoma, or cystadenocarcinoma.^[12,13]^ Simple hepatic cysts have typical imaging manifestations and are easy to be diagnosed clinically, but the imaging manifestations of hepatic cysts with intracystic hemorrhage are complex. Intracystic hemorrhage showed changes in cyst density, blood stratification in the cyst, or diffuse irregular hyperecho on imaging.^[10]^ Several neoplastic lesions of the liver can be confused with hepatic cysts with hemorrhage, including biliary cystadenoma, cystadenocarcinoma, and primary embryonal sarcoma.^[11]^ According to clinical and imaging data, it is sometimes difficult to distinguish hemorrhagic hepatic cysts from cystic malignant tumors when mural nodules with enhancement after intravenous administration of contrast material appear on CT or MRI examination.^[6,12]^ It is also worth to note that hemorrhagic cystic lesions are hyperintense on T1-weighted images, nonhemorrhagic cystic lesions are hypointense on T1-weighted images, and both cystic lesions are hyperintense on T2-weighted images. Nonhemorrhagic cysts are typically hypodense on CT, whereas hemorrhagic cysts can appear hyperdense in the acute phase and hypodense or mixed density after the acute phase.^[9]^ All of the above conditions may lead to a variety of changes in the imaging picture of simple hepatic cysts. These alterations make it difficult to diagnose hepatic cysts with hemorrhage on imaging with other liver diseases such as hydatid disease, biliary cystadenoma, or cystadenocarcinoma.^[12,13]^ Biliary cystadenocarcinoma, as a rare malignant epithelial tumor of the liver, lacks specific clinical manifestations. When the tumor increases and compresses the surrounding liver tissue and adjacent organs, abdominal pain, abdominal distension, jaundice, and abnormal liver function may occur. It is worth noting that although the above characteristics can also appear in patients with hepatic cysts and hemorrhage, the elevation of tumor markers (CA19-9, CA-125, etc) seems to be an important indicator to distinguish them. The occurrence of hepatic cystic metastases usually has a history of primary tumors, and the metastatic tumors vary in size and shape. Through imaging examination, patients can even find the presence of metastatic tumors in other parts of the patient, and the typical “bull’s eye sign” can appear under CT enhanced scanning. Tumor markers are also key to distinguish cystic metastases from hepatic cysts with hemorrhage. On preoperative examination, tumor markers, including alpha-fetoprotein, carcinoembryonic antigen, and serum carbohydrate antigen, were in the normal range; therefore, disease related to liver cancer was not considered in this patient’s diagnosis. Pyogenic liver abscess as an infectious disease, fever, chills and leukocytosis are important characteristics that distinguish it from hepatic cysts with hemorrhage. At the same time, due to the necrosis of the central area of the abscess and the formation of a peripheral edema zone, we usually see an air-fluid level and a peripheral edema zone without enhancement. Suppurative liver abscess was also excluded on the basis of the patient’s symptoms and imaging features. We sorted out the imaging features of hepatic cysts with hemorrhage and various other diseases (Table 1).

**Table 1:** The imaging features, differential points and the best imaging examination methods of various hepatic cystic diseases were summarized.

Disease	X-ray	Ultrasound	CT	MRI	Nuclear medicine	Optimal imaging method
Hepatic cysts with hemorrhage	–	1. Cyst separation 2. Internal echo is not uniform 3. Thickening of the cyst wall 4. With or without calcifications	1. CT scan: 1.1. Cyst wall nodules 1.2. With or without cyst wall calcification and fluid level 2. Enhanced CT scan: 2.1. The cyst wall and septation were slightly enhanced, and the cyst fluid was not enhanced	1. T1WI/T2WI: 1.1. Mildly heterogeneous signal (mixed intracystic fluid and blood) 1.2. With or without fluid levels 2. T1C+: 2.1. No reinforcement	–	Ultrasound CT
Simple hepatic cyst	–	1. Anechoic mass 2. Smooth border 3. Visible or invisible cyst wall 4. No or little separation 5. No nodules or calcifications in the cyst wall	1. CT scan: 1.1. Clear boundary and smooth cyst wall 1.2. The CT value was similar to that of water (‐10 to +10 Hu) 1.3. The cyst cavity is filled with liquid, without air liquid level 1.4. No cyst wall nodule sign and cyst wall calcification 2. Enhanced CT scan: 2.1. No obvious enhancement	1. T1WI: 1.1. Low signal 2. T2WI: 2.1. High signal 3. HeavilyT2W: 3.1. The signal of stagnant liquid is significantly enhanced 4. MRCP: 4.1. The cyst does not communicate with the intrahepatic bile duct	–	Ultrasound CT
Hepatic echinococcosis	1. X-ray examination: 1.1. *Echinococcus granulosus* had arc-shaped or signet ring-like peripheral calcification with 20% to 30% calcification, as shown on plain abdominal film 1.2. 50% of the multilocular echinococcosis had small calcifications 2. ERCP: 2.1. Hydatid cysts may communicate with the biliary tract, 84% in the intrahepatic bile duct, 9% in the common hepatic duct, 6% in the gallbladder, and 1% in the common bile duct	1. Echinococcosis granulosa: 1.1. Multiple asci surround the mother capsule 1.2. Well-defined hypoechoic mass 2. Multilocular echinococcosis: 2.1. Single or multiple hypoechoic lesions usually located in the right lobe of the liver 2.2. Irregular necrosis and small calcification, some with bile duct dilatation	1. CT scan: 1.1. Echinococcosis granulosa: 1.1.1. Large, single or multiple, well-defined, low density 1.1.2. Multiple peripheral daughter foci with slightly lower density than the parent foci 1.1.3. Arc-shaped signet ring calcification 1.1.4. Ring calcified wall 1.1.5. Dilatation of intrahepatic bile ducts 1.2. Multilocular echinococcosis: 1.2.1. Extensive, aggressive, low-density solid mass (14–40 Hu) 1.2.2. Regular boundary 1.2.3. Irregular shape of calcification 1.2.4. May cause primary or secondary tumors 2. Enhanced CT scan: 2.1. Echinococcosis granulosa: 2.1.1. Enhancement of cyst wall and septation 2.2. Multilocular echinococcosis: 2.2.1. The noncalcified part showed mild enhancement	1. T1WI: 1.1. Capsule (pericapsular): hypointense (fibrous component) 1.2. Mother capsule: usually moderate signal intensity, rarely high signal intensity 1.3. Ascus: signal is lower than that of mother 1.4. Calcification is not easy to identify 2. T2WI: 2.1. Capsule (pericapsular): Hypo signal (fibrous component) 2.2. Cystic lesions showed high signal intensity 3. T1C+: 3.1. The cyst wall and septation of *Echinococcus granulosus* were enhanced 3.2. Mild enhancement of the noncalcified portion of the multilocular echinococcosis 4. MRCP: 4.1. Some of the cysts communicated with the bile duct	1. ^99^Technetium sulfur gum: 1.1. Mass effect can be distinguished from focal fatty infiltration 2. Xe-^131^ 2.1. High lipid solubility 2.2. Radionuclides accumulate in the fatty deposition areas of the liver	CT
Cystic metastatic tumor of the liver	–	1. The shape is mostly regular, round or oval, with clear boundary and anechoic area in the interior 2. A few internal echoes are dense hypoechoic light spots, and the strip separation is slightly strong echo 3. Most of them had primary pancreatic cystadenocarcinoma and ovarian cystadenocarcinoma 4. No pseudopod-like extension to the surrounding tissue	1. CT scan: 1.1. The lesions were multiple, round or oval low-density shadows (CT value <20 Hu), with clear boundaries and difficult to display the cyst wall 1.2. The density of the capsule is uniform 1.3. Fluid level, necrotic foci, cyst wall nodules, and thick cyst wall and septa were seen in some cases 1.4. Often metastasis from cystadenocarcinoma and sarcoma (primary pancreatic, gastrointestinal, ovarian) 2. Enhanced CT scan: 2.1. Contrast-enhanced scan showed the metastatic cyst wall, which was thin and relatively homogeneous without significant enhancement 2.2. Mural nodules and septations were enhanced, and no enhancement was observed in the cystic cavity	1. T1WI: 1.1. Most of the lesions showed low signal, and a few showed mixed signal 1.2. The signal intensity of most lesions was homogeneous, and some lesions were heterogeneous. There was slightly high density-shadow inside, which was lower than the signal intensity of liver parenchyma 2. T2WI: 2.1. All lesions showed high signal intensity 2.2. Most of the signals were uneven, and the edge signals were slightly lower or wall nodules with slightly lower signals were seen 3. T1C+: 3.1. There was no enhancement in the arterial phase 3.2. Rim enhancement and wall nodule enhancement were observed in most lesions in portal and delayed phase 3.3. Some lesions showed mild peripheral enhancement, and the signal intensity was lower than that of liver parenchyma. Few lesions showed incomplete septa, and there was mild enhancement 3.4. Some intracapsular septations may be mildly enhanced	1. PET showed multiple hypermetabolic lesions	CT MRI
Biliary cystadeno-carcinoma	–	1. Large, well-circumscribed, polycystic anechoic lesion 2. Hyperechoic septa 3. The tumor grew nodular or papillary 4. Calcification can be seen in the cyst wall or septum, and fluid level can be seen in the cyst 5. The cystic fluid was anechoic or internally echoic	1. CT scan: 1.1. The lesions were large, well-circumscribed, and uniformly dense with water-like density 1.2. Cystic and hemorrhagic areas with uneven density within the lesion 1.3. Lobulated with nodu 1.4. Dilated bile ducts 2. Enhanced CT scan: 2.1. Polycystic neoplasms: 2.1.1. No enhancement of the cyst cavity 2.1.2. Intra-lesion septation, capsule and nodule enhancement 2.1.3. Enhancement of papillary vegetations 2.1.4. Calcification can be seen in the cyst wall and septation 2.1.5. With or without distant metastasis 2.2. Unilocular tumors: 2.2.1. No enhancement of the cyst cavity 2.2.2. Enhancement of capsule and papilliform vegetations 2.2.3. Calcification can be seen in the cyst wall	1. T1WI: 1.1. Viscous cystic fluid, increased signal 1.2. Dilute cystic fluid, decrease signal 1.3. Calcification of septation and cyst wall showed low signal 2. T2WI: 2.1. Viscous cystic fluid, decrease signal 2.2. Dilute cystic fluid, increased signal 2.3. Calcification of the septa and cyst wall was hypointense 2.4. Clear boundaries 3. T1C+: 3.1. Enhancement of capsule and septation can be seen	—	CT MRI
Pyogenic liver abscess	1. X-ray examination: 1.1. Atelectasis in the lower lobe of the right lung and right pleural effusion 1.2. Abdominal plain film showed hepatomegaly, intrahepatic gas accumulation, and air-fluid level	1. Pretumescent stage: clear boundary, uniform echo 2. Abscess formation stage: anechoic area with clear edge; The wall was thick, rough, and the inner wall was not smooth. There was a compartmental-like echo 3. In the absorption stage of abscess, the residual abscess showed patchy and cord-like hyperechogenicity	1. CT scan: 1.1. Simple pyogenic abscess: 1.1.1. Well-defined, round, low-density masses (0–45 Hu) 1.1.2. Multiple small abscesses may merge into a single abscess, which is called cluster sign 1.2. Complex pyogenic abscesses: 1.2.1. Peripheral low-density edema zone 1.2.2. Isodense abscess wall 1.2.3. Hypodense necrosis in the central area 1.3. Special signs: 1.3.1. Air-fluid level is present in <20% of abscesses 1.3.2. Large air–liquid level and liquid–liquid (necrotic tissue) level 2. enhanced CT scan: 2.1. Well-defined, round, low-density masses 2.2. Enhancement of abscess wall and septation 2.3. Atelectasis of the right lower lung and pleural effusion were observed 2.4. Nonliquefaction abscesses resemble hypervascularized tumors	1. T1WI: 1.1. Low signal 2. T2WI: 2.1. High signal intensity with surrounding hyperintense edema zone 3. T1C+: 3.1. No enhancement 3.2. Low signal block 3.3. Enhancement of the tumor wall 4. MRCP: 4.1. It is a specific test	1. Liver and bile sulfur glue scanning: 1.1. Round, low concentration area 1.2. The biliary tract was occasionally visible 2. WBC scan: 2.1. It has high specificity because leukocytes concentrate to form a concentration zone (hot zone)	CT

Cystic echinococcosis is a zoonotic disease caused by infection with *E granulosus*, which is caused by *E granulosus*, one of the smallest tapeworms in the family Taeniidae. During the life cycle of *E granulosus*, humans may occasionally become intermediate hosts through handling animal or egg-containing feces, plants, edible vegetables, undercooked fruit and drinking water containing eggs.^[14]^ Clinically, patients are usually asymptomatic at the beginning of infection, and the asymptomatic stage of infection can last for many years. Symptoms of hepatic cystic echinococcosis include epigastric pain, hepatomegaly, cholestasis, biliary cirrhosis, portal hypertension, and ascites.^[15]^ Serious complications include hydatid cyst rupture into the abdominal cavity leading to severe allergic reactions, secondary cystic echinococcosis, and cyst rupture into the biliary tract leading to cholangitis and cholestasis. Conventional imaging studies including ultrasound, CT scan and MRI scan can be used to detect hepatic echinococcosis.^[16]^ Serological examination is the main method for the diagnosis of hepatic echinococcosis.^[17]^ In very special cases, ultrasound-guided fine needle aspiration biopsy is essential for diagnosis.^[18]^ Studies have shown that the diagnosis of hepatic echinococcosis is usually based on the following criteria: the patient has a clear history of living in the epidemic area and the common clinical manifestations of hepatic echinococcosis (such as abdominal pain, chest pain, and fever), and the patient has a positive immunodiagnostic test and the presence of cystic space-occupying lesions in the liver by imaging examination.^[19]^ We comprehensively considered the patient’s history of living in the echinococcosis endemic area, abdominal ultrasound, and enhanced CT examination suggesting a huge cystic space-occupying lesion in the right lobe of the liver with a slightly increased density of cystic contents and positive anti-echinococcus immunoglobulin G antibody, and therefore, the cystic lesion in the right lobe of the liver was diagnosed as hepatic echinococcosis. Surgical treatment was performed. Although the patient recovered and was discharged from the hospital, this underscores the importance of imaging in the identification of hepatic hydatid disease and hepatic cysts with bleeding, even when the patient has a clear history of soresidence in an epidemic area and serologic testing is positive. The history of sobbed in the epidemic area can only be used as one of the diagnostic clues. Although the patient had mild tenderness in the right upper quadrant and accompanied by pain and discomfort in the right shoulder, this clinical manifestation was not obvious specificity. Serological test results can be used as one of the means of confirming hepatic echinococcosis, but their sensitivity is low and depends in part on the location of the cyst in the body and the cystic stage. Up to 20% of patients with solitary hepatic hydatid disease and up to 50% of patients with pulmonary hydatid disease may be seronegative at the time of diagnosis, whereas hydatid disease at other sites is usually seronegative.^[18]^ False positive results are most commonly cross-reactive and are often associated with cross-reactions to other tapeworm infections (*Echinococcus multilocularis*, *Taenia solium* cysticercosis) and some other parasitic diseases (schistosomiasis, liver fluxus, filariasis), as well as noninfectious diseases such as malignancies and cirrhosis.^[20]^ In conclusion, imaging studies are important in differentiating hepatic cystic lesions including hepatic cysts with hemorrhage from hepatic echinococcosis. By searching a large number of literatures, we summarized the image characteristics and differential points of hepatic cysts with hemorrhage and other hepatic cystic lesions in conventional imaging examinations (including X-ray, ultrasound, CT, MRI, and nuclear medicine). It is expected to provide more imaging support for the clinical diagnosis of hepatic cysts with hemorrhage.

## 4. Limitations

There were no indications of a parasite infection, such as *E granulosus*, despite the postoperative pathology results confirming that the patient had a hepatic cyst with intracystic bleeding. Four months following surgery, the patient’s abdomen CT scan revealed no signs of echinococcosis. After surgery, we intended to continue imaging to check for recurrence, however the patient did not continue follow-up at this facility due to financial constraints. As a result, the patient’s status following surgery could not be further understood due to the loss of follow-up.

## 5. Conclusion

In this case, we overemphasized the importance of the serological test results of hydatid disease and the patient’s travel history in the epidemic area, and failed to comprehensively analyze the imaging examination results of the patient’s hepatic cystic lesions. At the same time we underestimated the imaging examination in diagnosis of liver cyst and hemorrhage and to identify the liver can play a role in the cystic lesion. Therefore, this case raises the awareness of hepatic cyst and its complications, and emphasizes the importance of imaging examination for the diagnosis of hepatic cyst with hemorrhage. In addition, routine imaging images of hepatic cystic space-occupying lesions including hepatic echinococcosis should be analyzed more comprehensively before diagnosis to reduce the misdiagnosis of atypical hepatic cysts and their complications, which is very important for the choice of subsequent treatment for patients.

The authors declare that they have no known competing financial interests or personal relationships that could have appeared to influence the work reported in this paper.

## Author contributions

**Resources:** Yan He.

**Supervision:** Jun Wang.

**Writing – original draft:** Shuang Wang, Yan He, Xuchang He.

**Writing – review & editing:** Shuang Wang.
